# Samarium hexaboride is a trivial surface conductor

**DOI:** 10.1038/s41467-018-02908-7

**Published:** 2018-02-06

**Authors:** P. Hlawenka, K. Siemensmeyer, E. Weschke, A. Varykhalov, J. Sánchez-Barriga, N. Y. Shitsevalova, A. V. Dukhnenko, V. B. Filipov, S. Gabáni, K. Flachbart, O. Rader, E. D. L. Rienks

**Affiliations:** 1 Helmholtz-Zentrum Berlin für Materialien und Energie, Elektronenspeicherring BESSY II, Albert-Einstein-Straße 15, 12489 Berlin, Germany; 2 Institut für Physik und Astronomie, Universität Potsdam, Karl-Liebknecht-Straße 24/25, 14476 Potsdam-Golm, Germany; 3 Institute for Problems of Materials Science, National Academy of Sciences of Ukraine, Krzhyzhanovsky str. 3, Kiev, 03142 Ukraine; 4 Institute of Experimental Physics, Slovak Academy of Sciences, Watsonova 47, 04001 Košice, Slovakia; 5 Institut für Festkörperphysik, Technische Universität Dresden, 01062 Dresden, Germany; 6 Leibniz-Institut für Festkörper-und Werkstoffforschung Dresden, Helmholtzstraße 20, 01069 Dresden, Germany

## Abstract

SmB_6_ is predicted to be the first member of the intersection of topological insulators and Kondo insulators, strongly correlated materials in which the Fermi level lies in the gap of a many-body resonance that forms by hybridization between localized and itinerant states. While robust, surface-only conductivity at low temperature and the observation of surface states at the expected high symmetry points appear to confirm this prediction, we find both surface states at the (100) surface to be topologically trivial. We find the ${\bar{\varGamma }}$ state to appear Rashba split and explain the prominent $\bar X$ state by a surface shift of the many-body resonance. We propose that the latter mechanism, which applies to several crystal terminations, can explain the unusual surface conductivity. While additional, as yet unobserved topological surface states cannot be excluded, our results show that a firm connection between the two material classes is still outstanding.

## Abstract (web summary)

Samarium hexahoride is argued to be a topological Kondo insulator, but this claim remains under debate. Here, Hlawenka et al. provide a topologically trivial explanation for the conducting states at the (100) surface of samarium hexaboride; an explanation based on Rashba splitting and a surface shift of the Kondo resonance.

## Introduction

Interest in SmB_6_ has seen a resurgence as Dzero et al.^1^ proposed that the low-temperature Kondo insulating phase of this material could be topologically non-trivial. Material-specific theoretical treatments^2–5^ support this proposal by predicting a band inversion at the *X* point that gives rise to topological surface states at the ${\bar{\mathrm \Gamma }}$ and $\bar X$ points of the (100) surface. Experiments have since confirmed that only the surface remains metallic at low temperature^6–8^ and surface states have been observed at the predicted sites^9–16^. Could SmB_6_, after being identified as one of the first mixed-valence compounds and Kondo insulators^17^, now become the prototype of yet another phenomenon in solid-state research?

On the basis of high-resolution angle-resolved photoemission (ARPES) results, we will present a topologically trivial explanation for both surface states at the (100) surface. We find that the surface state in the Brillouin zone center lacks the attributes of a topological surface state. We further observe a surface shift of the Sm 4*f*-like intensity near the Fermi level that explains the presence of the metallic state at the $\bar X$ point.

## Results

### The ${\bar{ \varGamma }}$ state

**Fig. 1:**
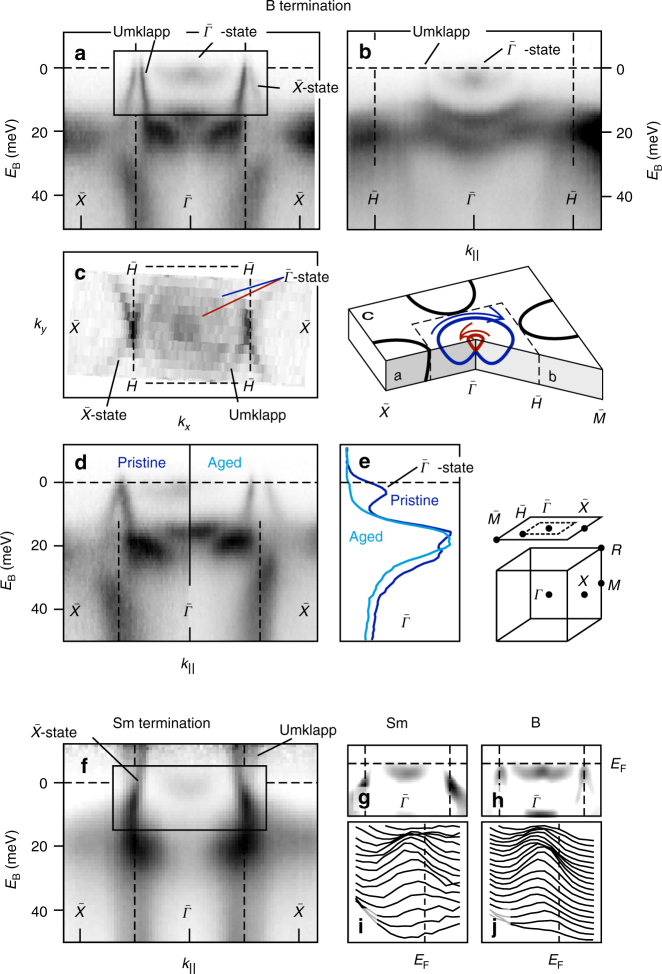
Massive nature of the ${\bar{\mathrm \Gamma }}$ state. Photoemission intensity (*I*) along **a** ${\bar{\mathrm \Gamma }}$ − $\bar X$ and **b** ${\bar{\mathrm \Gamma }}$ − $\bar M$ for the B-terminated surface. **c** The Fermi surface for B termination. **d** Photoemission intensity along ${\bar{\mathrm \Gamma }}$ – $\bar X$ on a pristine and aged B-terminated surface. **e** Photoemission spectra at ${\bar{\mathrm \Gamma }}$ of the pristine (blue) and aged (light blue) surface. **f** Photoemission intensity along ${\bar{\mathrm \Gamma }}$ – $\bar X$ on a Sm-terminated sample. Data in **f**, **g**, **i** obtained at 40 K to populate a larger fraction of the shallow ${\bar{\mathrm \Gamma }}$ state. Results have been divided by a Fermi-Dirac distribution. **g**, **h** Second derivative (d^2^*I*/d*E*^2^) of the photoemission intensity in the rectangular areas marked in **f**, **a**. **i**, **j** ${\bar{\mathrm \Gamma }}$ state represented as spectra for Sm and B termination. *hν* = 31 eV, *s*-polarization

We will first briefly address the surface state at ${\bar{\mathrm \Gamma }}$ (locations of high symmetry points in the surface Brillouin zone are indicated in Fig. 1) that was previously observed^10–13, 16, 18, 19^, but could not be characterized unambiguously on the basis of the available results. Here we can clearly resolve it in angle-resolved photoemission measurements on pristine B- and Sm-terminated surfaces (we find that samples can cleave with two well-defined terminations as is shown in Supplementary Fig. 1). For B termination, this state has a handlebar mustache-like dispersion (Fig. 1a, h, j). Several observations suggest that we are dealing with a trivial, Rashba-split surface state as known from noble metals^20^.

Firstly, while the shape somewhat resembles the topological surface state of Bi_2_Te_3_^21^, we do not find any evidence of the dispersion bending down to the lower parity-inverted bulk band, as required for a topological surface state. This is difficult to judge along ${\bar{\mathrm \Gamma }}$ – $\bar X$ (Fig. 1a) due to the intensity from the second surface state centered around $\bar X$ and its umklapp intensity (we observe the effect of a superstructure with 2*a* periodicity for either termination, as discussed in the Supplemental Material). Along the ${\bar{\mathrm \Gamma }}$ – $\bar H$ – $\bar M$ direction shown in Fig. 1b, however, a downturn would not be obscured by the intensity of the $\bar X$ state. In Fig. 1b, we only observe the umklapp intensity of the $\bar X$ state and no connection from the Rashba-split ${\bar{\mathrm \Gamma }}$ state to the lower parity-inverted bulk band, which is expected to be the lower band of the Sm 4*f*-like manifold^22^.

Secondly, aging of the sample at low temperature gives rise to *p*-doping of the surface electronic structure (discussed in more detail in Supplementary Fig. 4). This effect appears to push the ${\bar{\mathrm \Gamma }}$ state entirely above *E*_F_, shown in Fig. 1d and e. Its traceless disappearance is another indication that the ${\bar{\mathrm \Gamma }}$ state we observe is of trivial nature, as a topological surface state necessarily spans the band gap by crossing the Fermi level (*E*_F_) an odd number of times between time-reversal invariant momenta^1–5^.

Finally, the ${\bar{\mathrm \Gamma }}$ state appears as a single parabola on the Sm-terminated surface, as shown in Fig. 1f, g, and i. This result strongly hints at Rashba splitting, because this effect relies on the gradient of the crystal potential perpendicular to the surface. Given the polar nature that bulk-truncated surfaces with pure B or Sm termination would have, a difference in surface potential gradient is to be expected for the different terminations. In Supplementary Fig. 2, we show that the B 2*p* valence band displays a clear binding energy shift between terminations, which supports a termination dependence of the surface potential. We point out that a fairly small difference in gradient would suffice given the very high effective mass (m^*^ ~ 17*m*_e_). The size of Rashba splitting is proportional to the mass: Δ*k*_||_ = m^*^α_R_/*ħ*^2^, where *α*_R_ is the Rashba parameter that contains the spin–orbit interaction and potential asymmetry. When we interpret the ${\bar{\mathrm \Gamma }}$ state at the B-terminated surface as a Rashba-split free-electron-like state we obtain a very modest *α*_R_ of (3.5 ± 0.1) × 10^−12^ eV m. For reference, *α*_R_ = 3.3 × 10^−11^ eV m for the Rashba-split surface state of Au(111)^20^. We will see in the next section, however, that there is a considerable Sm 4*f* binding energy difference between the outer bulk-coordinated layers on B- and Sm-terminated samples. The precise correspondence in binding energy of the ${\bar{\mathrm \Gamma }}$ features on B-and Sm termination (*E*_B_ = 2.3 ± 0.6 meV) could therefore be coincidental. Its large effective mass, however, clearly shows that the ${\bar{\mathrm \Gamma }}$ state stems from a bulk 4*f* state and not from a B 2*p* state as suggested by Zhu et al.^23^ We finally add that a similarly wide and shallow ${\bar{\mathrm \Gamma }}$ state has been observed by Xu et al.^18^

In conclusion, while we cannot rule out the existence of a topological surface state inaccessible to photoemission, we only find evidence of topologically trivial features at ${\bar{\mathrm \Gamma }}$. As noted before^19^, the absence of a topological surface state at ${\bar{\mathrm \Gamma }}$ would have far-reaching implications: Because there are two $\bar X$ points in the surface Brillouin zone, the ${\bar{\mathrm \Gamma }}$ state is required to arrive at an odd number of topological surface states and, hence, a strong topological insulator. Moreover, its absence would directly render SmB_6_ a trivial insulator, as the cubic symmetry prohibits a weak topological insulator phase^4^. In the following we will further present a trivial explanation for the widely observed^9–16^ surface state at $\bar X$.

### The $\bar X$ state

**Fig. 2:**
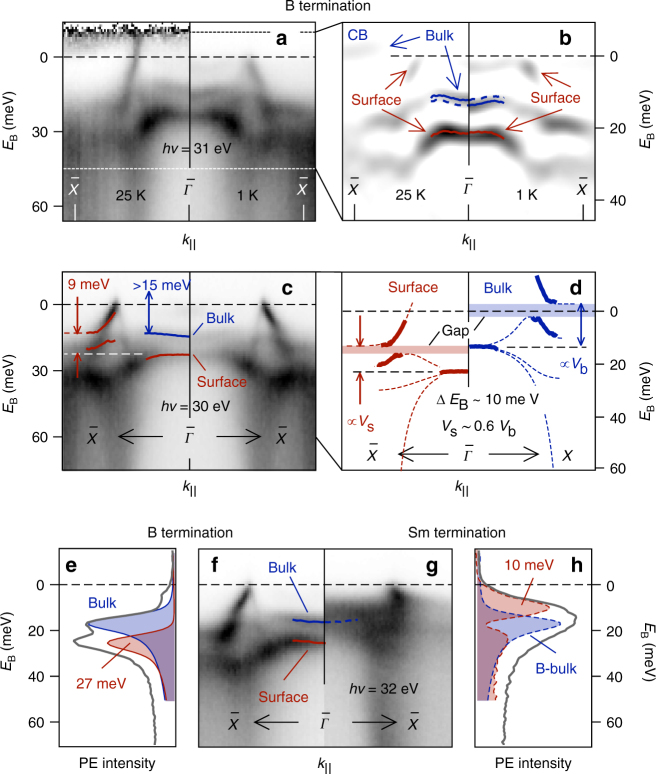
Surface and bulk contributions to the photoemission intensity. **a** Evolution of bulk and surface features as a function of temperature. **b** Second derivative (d^2^*I*/d*E*^2^) of the photoemission intensity shown in **a**. Solid lines indicate maxima of energy distribution curve fits. Dashed lines in **b** indicate the position of the maxima in the other half of the graph. **c** Photoemission intensity of the B-terminated surface. Solid lines indicate position of maxima from fits of the Doniach–Sunjic function to energy distribution curves. **d** Surface features from **c** are reproduced as solid red lines. Dashed lines are guides to the eye, connecting the energy distribution curve maxima in a way similar to the calculated band structure^3^. The estimated bulk structure (blue) is obtained by scaling and shifting of the surface components (see text). Filled areas indicate the width and position of the estimated surface and bulk band gaps. **f**, **g** Photoemission intensity for B- and Sm-terminated surfaces. Solid lines indicate maxima of energy distribution curve fits. Dashed line in **g** indicates the position of the bulk component for B termination. **e**, **h** Energy distribution curves for B-and Sm termination. Filled curves in **e** indicate fits of Doniach–Sunjic functions. Blue dashed curve in **h** indicates position of the bulk component from B termination (intensity arbitrarily scaled), red dashed curve shows the residual (surface) spectral weight when the bulk component is subtracted from the spectrum. All results obtained with *p*-polarization

**Fig. 3:**
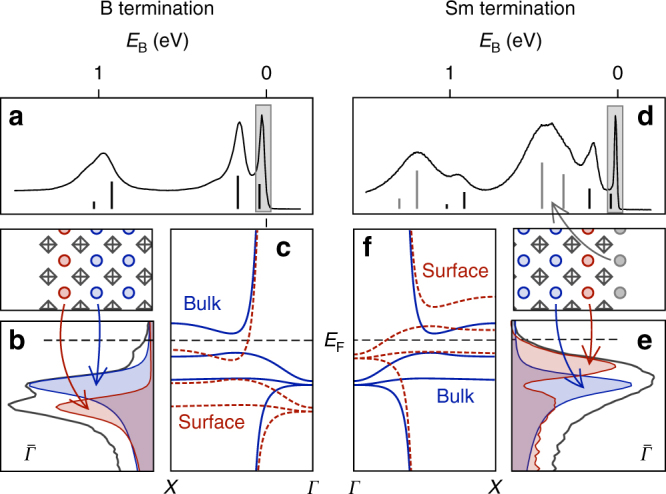
Origin of the $\bar X$ state. **a** Photoelectron spectrum (*hν* = 70 eV) on a B-terminated sample. **b** Electron distribution curve at ${\bar{\mathrm \Gamma }}$ obtained with *hν* = 32 eV. A tentative assignment of the different components is given in the sketch of a bulk-truncated B-terminated surface. **c** Illustrates how a displacement of the hybridization region by the observed many-body resonance shift would yield a metallic surface feature around $\bar X$. Solid (blue) curves show the band structure adopted from Lu et al.^3^ Dashed (red) curves show the effect of a rigid displacement of the hybridization region to higher binding energy. **d**–**f** Corresponding results for the Sm-terminated surface

Both Min et al.^14^ and Denlinger et al.^15^ have reported that the bulk conduction band (CB) moves above *E*_F_ with decreasing temperature, thereby confirming the opening of the hybridization gap. We observe the same effect as shown in Fig. 2a and b, where the spectral weight around *E*_F_ at $\bar X$ visible at 25 K disappears upon cooling to 1 K. Beyond this previously reported result, we can resolve how the upper occupied 4*f*-like band around ${\bar{\mathrm \Gamma }}$ simultaneously moves down by ~1.5 meV. This feature can thus be assigned to the bulk valence band that moves to higher binding energy as the hybridization gap widens. Interestingly, there is an identically dispersing band about 10 meV below it that we can interpret as a surface feature, because it changes markedly upon adsorption of gases. Results from both prolonged exposure to residual gas species and deliberate exposure to O_2_, are given in Supplementary Fig. 3. We note that bulk and surface components can be resolved at ${\bar{\mathrm \Gamma }}$ because, according to the band structure calculated by Lu et al.^3^ (reproduced in part in Fig. 3c and f), the triad of shallowest quasiparticle bands is degenerate at Γ (we note that the data in Fig. 2a and b are obtained with a photon energy of 31 eV, not exactly at *hν* = 26 eV where the bulk Γ point is expected. Measurements at 26 eV show the same effect although the matrix element near *k*_||_ = 0 is strongly reduced). In the framework of a Kondo insulator, the heavy states that appear around *E*_F_ should be interpreted as part of the many-body (Kondo) resonance that forms upon 5*d*–4*f* hybridization. We can thus conclude that the surface many-body resonance appears shifted to higher binding energy by about 10 meV with respect to the bulk. We attribute this shifted feature to Sm covered by at least one B layer (as sketched in Fig. 3). Like Denlinger et al.^19^, we assign the much larger shift (>300 meV) seen in the spectra in Fig. 3d and Supplementary Fig. 1b, to under-coordinated Sm. The latter assignment is in line with both surface shifts observed for other rare earth compounds, and the observed reactivity of this species^23^. Although small, the 10 meV surface shift we find for B termination is crucial, because it still considerably exceeds the ~3.5 meV activation energy found in transport measurements^6, 24, 25^. This activation energy is often attributed to (as yet unidentified) states inside a wider 15–20 meV bulk hybridization gap^26, 27^, and photoemission results are generally regarded to support this notion of a large bulk gap. As we will see shortly, however, the results depend strongly on the sample termination in this regard as well. An interpretation that correctly accounts for surface and bulk contributions is therefore required. Here we will argue that the hybridization gap could very well be on the order of the transport activation energy: in Fig. 2c, we can identify several features that, on the basis of their discreteness and photon energy indepedence, we attribute to the surface (supported by results in Supplementary Fig. 5). These correspond reasonably well to the LDA + Gutzwiller band structure proposed by Lu et al.^3^, in which two out of three 4*f* bands hybridize with the 5*d* band. When we interpret the shallowest surface feature in Fig. 2c as the surface analog of the bulk conduction band, we can see that it lies only ~9 meV above the main surface intensity at ${\bar{\mathrm \Gamma }}$. This suggests that hybridization at the surface is weaker, since the bulk conduction band lies at least 15 meV above the bulk intensity at ${\bar{\mathrm \Gamma }}$. With this result and the 10 meV binding energy shift between bulk and surface discussed above, we can provide an estimate of the bulk band structure along the Γ − *X* direction. By scaling the dispersion of the surface features by a factor of 15/9 and shifting it towards *E*_F_ by 10 meV, we get the structure shown in blue in Fig. 2d. The apex of the lower hybridizing band approaches *E*_F_ within a few meV (albeit only in a small fraction of the Brillouin zone) and we arrive at an indirect bulk band gap on the order of the activation energy found in transport measurements. We have not been able to observe the proposed bulk valence band, which would confirm this suggestion directly. We attribute this to (i) insufficient *k*_⊥_ resolution (shown in Supplementary Fig. 6c) and (ii) the omnipresence of the $\bar X$ surface state and its umklapp intensity (Supplementary Fig. 5b). In summary, we propose that the $\bar X$ state arises due to a shift of the many-body resonance that exceeds the bulk hybridization gap.

Analogously, on Sm-terminated samples we seem to observe a surface shift of the many-body resonance as well: a direct comparison between Sm and B-terminated samples is given in Fig. 2e–h. It reveals that the hybridization gap seems smaller for Sm termination. As the only difference between Fig. 2f and g is the termination, we can immediately attribute the extra intensity around ${\bar{\mathrm \Gamma }}$ at lowest binding energy for Sm termination to the surface. Unfortunately, bulk and surface components cannot be resolved as neatly as for B termination. If we, however, superimpose the bulk component (obtained from B termination) on the spectrum in Fig. 2h, a lot of spectral weight with a binding energy of about 10 meV remains that must be of surface nature. We can therefore conclude that Sm termination induces a surface shift near *E*_F_ of similar magnitude as B termination, but of opposite sign. We note that this ~−10 meV shift is most likely due to Sm atoms with bulk coordination, since the under-coordinated Sm on this termination gives rise to the large (>300 meV) 4*f* shift. We can again support this assignment by means of O_2_ adsorption experiments, details are given in Supplementary Fig. 3.

Independent experimental evidence of a termination dependence of the many-body resonance energy comes from the scanning tunneling microscopy studies by Yee et al.^28^ and Rößler et al.^29^ Both groups observe peaks in differential conductance spectra at either 28 or 8 meV on differently terminated, well-ordered parts of the surface. These maxima correspond very well to the surface components we observe for B and Sm termination shown in Fig. 2e and h. We do note that both groups attribute the observed terminations to structures that are incompatible with our spectroscopic results. This topic is discussed in more detail in the Supplemental Material.

In summary, the many-body resonance appears to be shifted energetically by an amount that exceeds the bulk band gap at both the B- and Sm-terminated surface.

An interpretation of the $\bar X$ state as a shifted, surface localized *d*–*f* hybrid implies that it is not confined to the bulk hybridization gap. Instead, it must be part of an energetically wider two-dimensional feature. We can confirm this by means of photon energy dependent measurements that can reveal the dispersion of electronic states perpendicular to the surface, i.e., along *k*_⊥_. We find clear evidence of a two-dimensional structure at a binding energy well beyond the bulk hybridization gap (results are given in Supplementary Fig. 6). This result supports our interpretation of the $\bar X$ state as a surface *d*–*f* hybrid with an energetically displaced hybridization region. It appears, however, to invalidate the premise that the $\bar X$ state is an independent in-gap state that forms the basis for the interpretation of the feature as topological. We attribute the *k*_⊥_-independent signal to the surface 4*f* share in the *d*–*f* hybrid, as only this component is both spatially and energetically sufficiently distinct from its bulk counterpart. The fact that the two-dimensional, *k*_⊥_ independent signal persists 50 meV below the 4*f* multiplet maximum (as seen in Supplementary Fig. 6d), indicates that 4*f* character is still large enough to be detected at this energy. This means the 4*f* state density profile must have a substantial width around the central energy of the many-body resonance. As a consequence, a finite fraction of the 4*f* state density profile remains above *E*_F_ even when the *d*–*f* hybrid is shifted to higher binding energy by ~10 meV. Resonant photoemission experiments presented in Supplementary Fig. 7 confirm that the surface Sm at a B-terminated surface remains in a mixed *f*^5^, *f*^6^ configuration.

A shift of the band structure as suggested in Fig. 3c implies a higher occupancy of Sm states at the surface as the *f*-like part of the conduction band around $\bar X$ is pushed below *E*_F_. As the $\bar X$ pockets cover just over a quarter of the surface Brillouin zone, we can estimate the charge on the surface Sm to be reduced by a little more than half an electron from the bulk value. To understand why the charge on the surface Sm layer would be lowered, we recall that the (100) surfaces of the bulk-truncated crystal are polar. In a perfectly bulk-truncated system, the surface electrostatic energy would diverge. Divergence is avoided by halving the charge on the outer layer (or by relaxation and reduction of charges on a larger number of layers). This led Zhu et al.^23^ to suggest that the charge on the outer B layer will be reduced, which gives rise to a metallic B 2*p* surface state. We can now see why this scenario is very unlikely: not only do the highest occupied B 2*p* states lie about 1 eV below *E*_F_, they also have considerable bandwidth (Supplementary Fig. 2). The Sm *d*–*f* hybrid on the other hand, will respond to potential changes much more swiftly: a 10 meV shift of the many-body resonance suffices to reduce the charge substantially, due to its 4*f*-like character near *E*_F_. We therefore propose that the charge reduction of the outer Sm layer by approximately half an electron is a result of the surface potential that builds up as a consequence of the polar termination.

At the Sm-terminated surface, the outer, under-coordinated Sm layer appears to assume a similar role: the shallowest multiplet from this species appears at a binding energy of 300 meV, from which we can conclude that the surface Sm layer has a pure *f*^6^ configuration. This suggests that the charge at the outer Sm layer is reduced to 2+ at this surface as well. It is more difficult to account for the first bulk-coordinated Sm layer at the Sm-terminated surface, which we argue is responsible for the shifted many-body resonance. Obviously, rigidly shifting the calculated band structure by Lu et al.^3^, as sketched in Fig. 3f, does not yield a satisfactory description of the results: a metallic electron-like band around the $\bar X$ point is observed experimentally. The uncertainty about the band dispersion prevents us from giving an estimate of the charge contained in this layer. We note, however, that the surface spectral weight profile displayed in Fig. 2h appears to have a bimodal distribution around the bulk component. This indicates that the 4*f* filling is not reduced by as much as suggested by a −10 meV shift.

In this way, we can understand some of the differences between B and Sm termination: the $\bar X$ state appears heavier for Sm termination as can be seen by comparing Fig. 2f and g. This is to be expected for a band with more 4*f* character than the more 5*d*-like conduction band that forms the $\bar X$ feature on B-terminated surfaces. The termination-dependent binding energy of the surface 4*f* component can also explain some of the variance in published photoemission results: Denlinger et al.^15^ observed a shallow feature with 4*f* character at the $\bar H$ point that was not reported in prior studies. We can now attribute this to an Sm-rich termination.

We conclude that for two different terminations of the clean crystal, a small shift of the many-body resonance is induced in apparently bulk-coordinated Sm layers. We argue that this mechanism can explain the robust surface conductivity observed in this material: any termination for which the bulk potential is not precisely matched in the outer bulk-coordinated layer(s) of the SmB_6_ crystal, will induce a shift of the many-body resonance. As soon as this shift exceeds the small band gap, the requirement that makes the bulk insulating is no longer met and metallic fringes form. We stress that this mechanism does not involve the emergence of a robust metallic state, but instead relies on the fairly easy destruction of the frail Kondo insulating state of the bulk. The fact that the many-body resonance shift is induced in bulk-coordinated Sm—i.e., in chemically pure SmB_6_—further guarantees the presence of such a layer regardless of the termination or nature and thickness of a capping film. The only requirement is the magnitude of the many-body resonance shift.

To support the notion that the natural oxide of SmB_6_ could fulfill this requirement, we have studied the interface with an ultrathin oxide layer. Such a film forms upon room temperature oxidation of the Sm-terminated surface (details are given in Supplementary Fig. 8). We find that this third termination gives rise to another many-body resonance shift and a matching Fermi-wave vector (*k*_F_) for the $\bar X$ state.

## Discussion

We would finally like to point out that while we find strong evidence of a trivial origin of the surface states at the ${\bar{\mathrm \Gamma }}$ and $\bar X$ points, our ARPES results remain fully consistent with almost all published data: we can attribute the variation in ${\bar{\mathrm \Gamma }}$ features reported in the literature^10–13, 16, 18, 19^ to the use of various (mostly unspecified) crystal terminations and different stages of sample aging. We can also explain the much more consistent reports on the appearance of the $\bar X$ state^9–16^. The size of the Fermi surface contours around $\bar X$ is fairly termination independent, because the associated Sm 4*f* energy variation is very small with respect to the Sm 5*d* bandwidth. Our trivial interpretation of the $\bar X$ state appears to conflict only with the experimental results of Xu et al.^13^ who have reported the helical spin texture expected for a topological surface state. We point out, however, that the possibility of a spin-polarized Rashba-split state of considerable extent in *k*-space, as we observe around ${\bar{\mathrm \Gamma }}$, has not been considered in their interpretation. We further note that it is not clear why spin-resolved measurements on YbB_6_ by the same group yield a topological spin texture for the surface state at $\bar X$^30^, whereas a strong case can be made that the dispersion is that of a trivial state^31, 32^. Possibly, spin-polarized emission from the surface could have a different origin, such as the emergence of surface magnetism^33^.

Although our results thus render SmB_6_ a trivial surface conductor, we stress that this does not rule out that it is a topological insulator as well. We note that, to ultimately settle the question whether topological surface states exist in SmB_6_, it will be necessary to terminate the crystal in such a way that the hybridization gap persists at the interface. Only in this way one can gain an unobscured view.

## Methods

### Crystal growth and characterization

SmB_6_ powder has been synthesized by borothermal reduction of Sm_2_O_3_ with metallic B powder under vacuum at 1900 K. The starting components were amorphous natural B and Sm_2_O_3_ with a purity of 99.9 and 99.996%, respectively. The resulting SmB_6_ powder was pressed to rods that were sintered under vacuum at 2000 K. The sintered rods of ~8 mm diameter and 60 mm length then have been used for single crystal growth with an inductive, crucible-free floating zone technique under 0.4 MPa Ar pressure. Single crystals with a diameter of typically 6 mm and a length of up to 40 mm were grown using [100] oriented seeds. The growth procedure was repeated twice to remove any porosity from the starting feed rods. During the first passage the crystallization rate was 1 mm/min, which gave the primary crystal without pores. At the end of the first passage the melting zone was frozen and the crystal was zone-melted again to the opposite side with a crystallization rate of 0.4 mm/min while rotating the crystal with 5 rpm. Powder X-ray diffraction patterns of the crushed SmB_6_ crystal revealed the presence of SmB_6_ only. Moreover, Laue back scattering patterns from both ends of the crystal show a single crystal with [100] orientation. The crystal employed for the present ARPES experiments showed a residual resistance of *R*(1 K)/*R*(300 K) = 5.3 × 10^4^. Oriented slabs with length up to 5 mm and typical cross section of 1 × 1 mm^2^ have been cut for the ARPES experiments. The orientation was again verified using X-ray Laue diffraction which gave sharp reflections and did not show any twinning. Samples were cleaved in ultrahigh vacuum (<10^−10^ mbar) at temperatures below 40 K.

### Photoemission spectroscopy

Photoemission experiments were performed with the 1^3^ ARPES end-station at the UE112–PGM2b beamline of BESSY II. The experimental geometry in this system is as follows: With the central axis of the analyzer lens and the polar rotation axis of the sample defined as the *x* and *z* axes of a spherical coordinate system, the photons arrive from the direction with *θ* = 45° (azimuth) and *ϕ* = 84° (polar). The entrance slit of the hemispherical analyzer is placed parallel to the *z* axis. Data at *hν* = 31 eV were obtained with an energy resolution of 3 meV. A sample temperature of 1 K is used unless indicated otherwise. *k*_⊥_ values are calculated assuming a free-electron final state using an inner potential of 14 eV.

### Data availability

The data that support the findings of this study are available from the corresponding author upon reasonable request.

## Electronic supplementary material

Supplementary Information

Peer Review File
