# Supplementary material for: Samarium hexaboride is a trivial surface conductor
Source: Nat Commun. 2018 Feb 6;9:517. doi: 10.1038/s41467-018-02908-7 (PMC5802797; doi:10.1038/s41467-018-02908-7)
Supplement: Supplementary file 1 — Supplementary Information [file 41467_2018_2908_MOESM1_ESM.pdf]

Supplementary Information

Samarium hexaboride is a trivial surface conductor

P. Hlawenka *et al*.

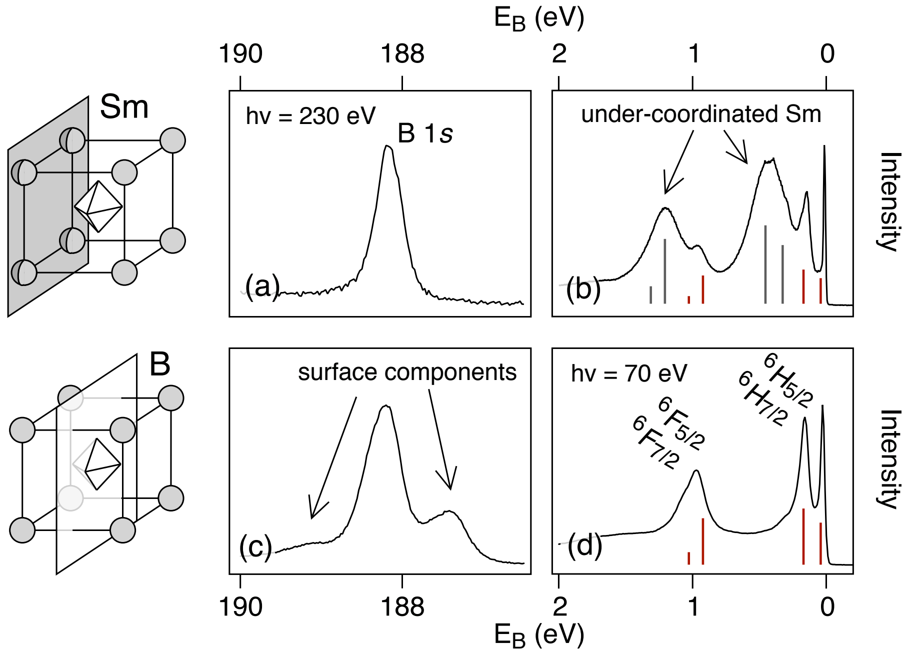

Supplementary Fig. 1. Surface terminations. The crystal is found to cleave with two different terminations that give distinct photoemission spectra: An apparently purely Sm terminated surface which is characterized by a simple B 1*s* spectrum (a) and a more complex 4*f* valence band spectrum. The part which contains the *f*-orbital multiplet due to the $4f^6 \rightarrow 4f^5$ photoemission transition (b) features broad additional surface components shifted to higher binding energy by approximately 300 meV. The other termination apparently exposes B atoms exclusively, evidenced by the simple 4*f* spectrum (d) and the more complex B 1*s* spectrum with additional surface components on either side of the main feature. Similar variations of the valence band spectrum have been observed by Denlinger and coworkers [1]. All results presented in the present work were obtained with surfaces of which the size of regions with a single termination exceeds the size of the synchrotron beam profile (~ 250 μm). Cleaved samples occasionally expose a surface that does not exhibit contrast in angle resolved photoemission at $h\nu < 40$ eV. We suggest that this can be attributed to the unordered (filamentary) terminations observed by Yee, He et al. [2]. Bars in (b,d) indicate calculated intensity for the $f^6 \rightarrow f^5$ (Sm^{2+}) photoemission transition [3].

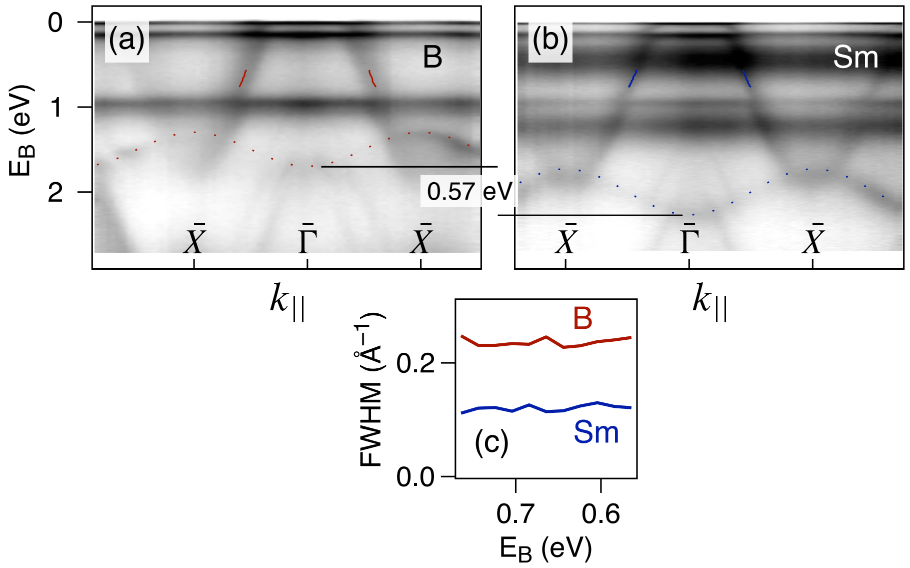

Supplementary Fig. 2. Termination dependence of the B $2p$ valence band. Photoemission intensity showing the top of the B $2p$ valence band along $\bar{\Gamma} - \bar{X}$ for (a) a B terminated and (b) Sm terminated sample ($h\nu = 70$ eV; p-polarization). Dots highlight the dispersion of a shallow B $2p$ band. The binding energy of this feature shows a termination dependence of > 0.5eV, confirming the assertion that the surface potential differs between terminations, discussed in the main text in relation to possible Rashba splitting of the $\bar{\Gamma}$ surface state. We can also conclude from the figure that the top of the B $2p$ bands remains about 1 eV below E_F for either termination. This means that the charge of the B surface layer is not reduced with respect to the bulk, as predicted by Zhu et al. [4]. Solid lines in (a,b) further indicate maxima of Lorentzian fits to momentum distribution curves. The full width at half maximum of these Lorentzians fit to the $5d$-like sections are given in (c). We attribute the larger width for B termination to more 3-dimensional character (corresponding to a more filled, partially resolved bulk band). Since we argue that the $d - f$ hybrid acquires partial two-dimensional character due to the contribution of $4f$-like components that are locally (i.e., at the surface) displaced in energy, the $d - f$ hybrid at the Sm terminated is more two-dimensional due to the presence of an additional set of f-orbital multiplet lines at which mixing occurs.

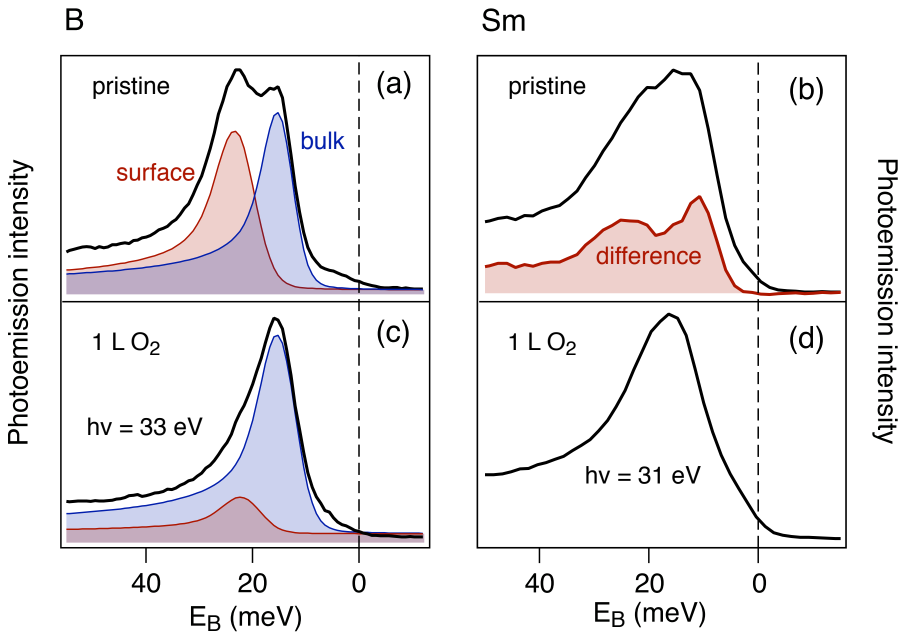

Supplementary Fig. 3. Effect of low temperature O_2 and residual gas exposure. We have studied the effect of O_2 and residual gas adsorption to identify which components in the photoemission spectra can be attributed to the surface. For the B terminated sample, we find that O_2 exposure mostly affects the 4*f*-like component with higher binding energy, as seen by comparing spectra at $\bar{\Gamma}$ from a pristine (a) and O_2 exposed (c) sample. Filled red and blue curves are the two main components from a least squares fit with Doniach-Sunjic functions (a third minor component to account for the intensity of the $\bar{\Gamma}$ surface state close to E_F is not shown). This result forms the basis of our assignment of the shallower 4*f*-like level to the bulk and the higher binding energy component to the surface. In the spectra from the Sm terminated surface, shown in (b) and (d), there is not such a clear separation between components that can be attributed to surface and bulk. We can, however, identify surface features by means of the difference between the pristine and O_2 exposed spectra, given by the filled red curve in panel (b). The difference highlights the parts of the spectrum most affected by surface modification and corresponds quite well to the estimated surface contributions in (main text) Fig. 2(h) obtained by subtraction of the bulk component (from B termination) from the pristine Sm terminated spectrum. All results obtained with *p*-polarization.

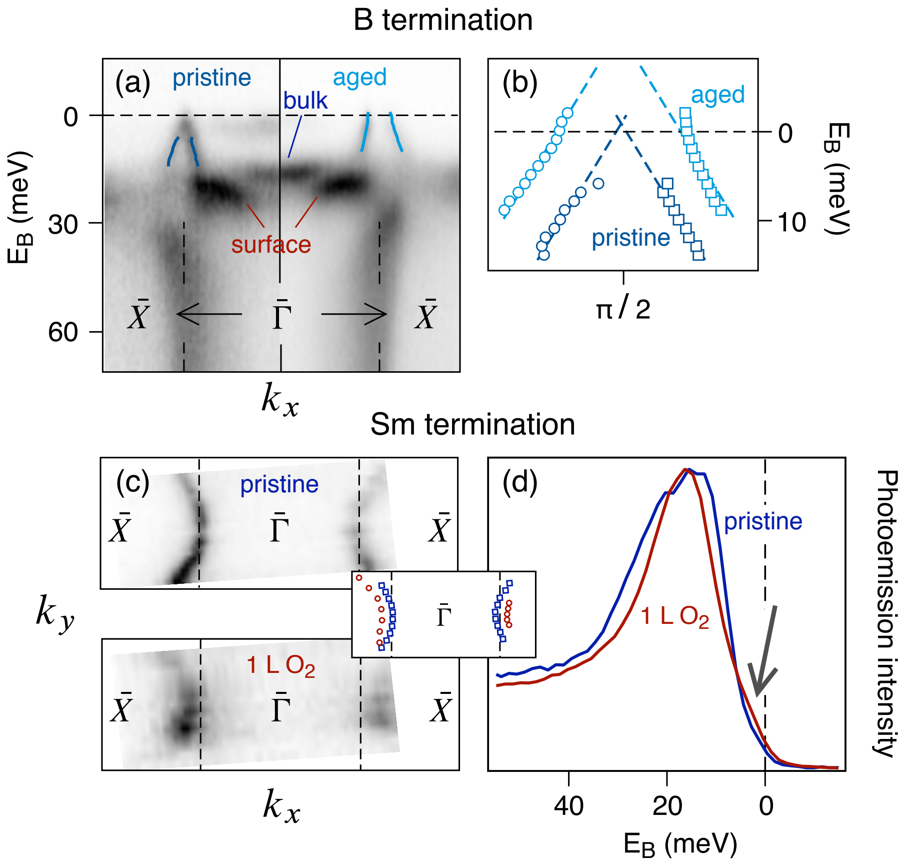

Supplementary Fig. 4. Binding energy of the surface many-body resonance and the size of the $\bar{X}$ Fermi surface contour. After mild exposure to the ultrahigh-vacuum (UHV) residual gas we can observe a small shift of the surface 4*f*-like component's binding energy. (a) The result of exposing a B terminated surface to the UHV residual gas at low temperature ($T < 25$ K) over a 24 hour period. The binding energy of the shallower 4*f*-like component around $\bar{\Gamma}$ is not noticeably affected, whereas the deeper component labeled *surface* shows a clear shift towards the Fermi level ($h\nu = 31$ eV; *s*-polarization). Simultaneously, the $\bar{X}$ state also shifts up (causing the Fermi surface contour around $\bar{X}$ to shrink) as shown by the increasing separation between the original and back-folded intensity. Solid blue curves connect the maxima from momentum distribution curve fits. The energy displacement between the linear fits to momentum distribution curve maxima in (b) amounts to ~ 10 meV. This shows a direct link between the binding energy of the surface many-body resonance and the size of the $\bar{X}$ contour, in agreement with the interpretation of the $\bar{X}$ state as the surface $d - f$ hybrid. A similar effect can be observed for the Sm terminated surface: (c) Exposure to 1 L of O_2 at $T = 15$ K leads to a decrease in size of the $\bar{X}$ contour. The inset shows the fitted maxima of the Fermi surface contours in (c). This change can also be linked to an apparent *p*-doping of the surface electronic structure. The energy distribution curves at $\bar{\Gamma}$ in (d) reveal that the shallowest 4*f* intensity —assigned to the surface— shifts to even smaller binding energy and gives rise to a shoulder almost crossing E_F, indicated by the arrow in (d). Results in (c,d) obtained with $h\nu = 33$ eV; *p*-polarization.

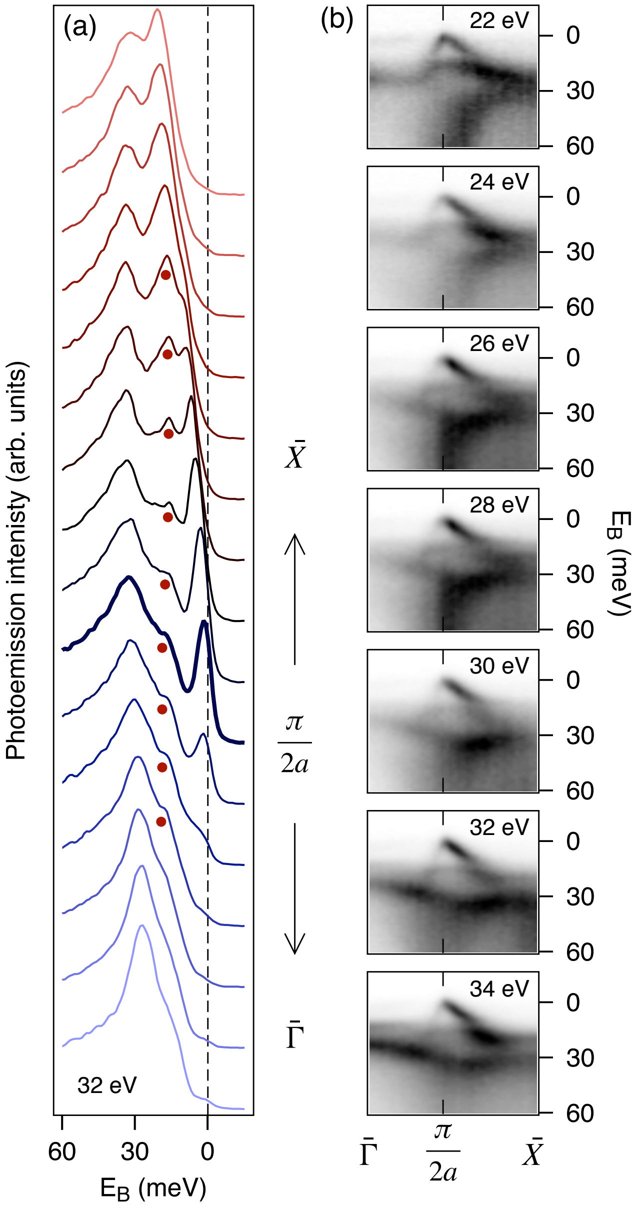

Supplementary Fig. 5. Surface band structure. We can observe a number of fairly discrete, quasiparticle-like features in the photoemission intensity along the $\bar{\Gamma} - \bar{X}$ direction near $k_{||} = \pi/2a$ for B terminated samples (b). We attribute these features to the surface because, apart from some intensity variation, they appear $h\nu$-independent. The photon energy dependent photoemission data in (b) span the $k_{\perp}$ range from 3.8 to 4.5 π/a. The photoemission intensity at $h\nu = 32$ eV is further presented as a set of energy distribution curves in (a). The $\bar{X}$ state, that we interpret as part of the surface conduction band, can be seen to cross E_{F} near $k_{||} = \pi/2a$. We interpret the maximum just below it (marked with red dots) as the surface valence band (compare with the model in Fig. 2(d) in the main text). The dispersion of this feature (and the $\bar{X}$ state just above it) has been determined by means of Lorentzian fits to energy distribution curves. These are shown in Figs. 2(c) and (d) in the main text as solid red lines. The smallest direct band gap between this band and the $\bar{X}$ state (i.e., between the surface valence and conduction bands) is approximately 7 meV. We cannot provide an estimate of the indirect surface band gap, because we can no longer resolve the position of the surface conduction band closer to $\bar{X}$. All data obtained with p-polarized light.

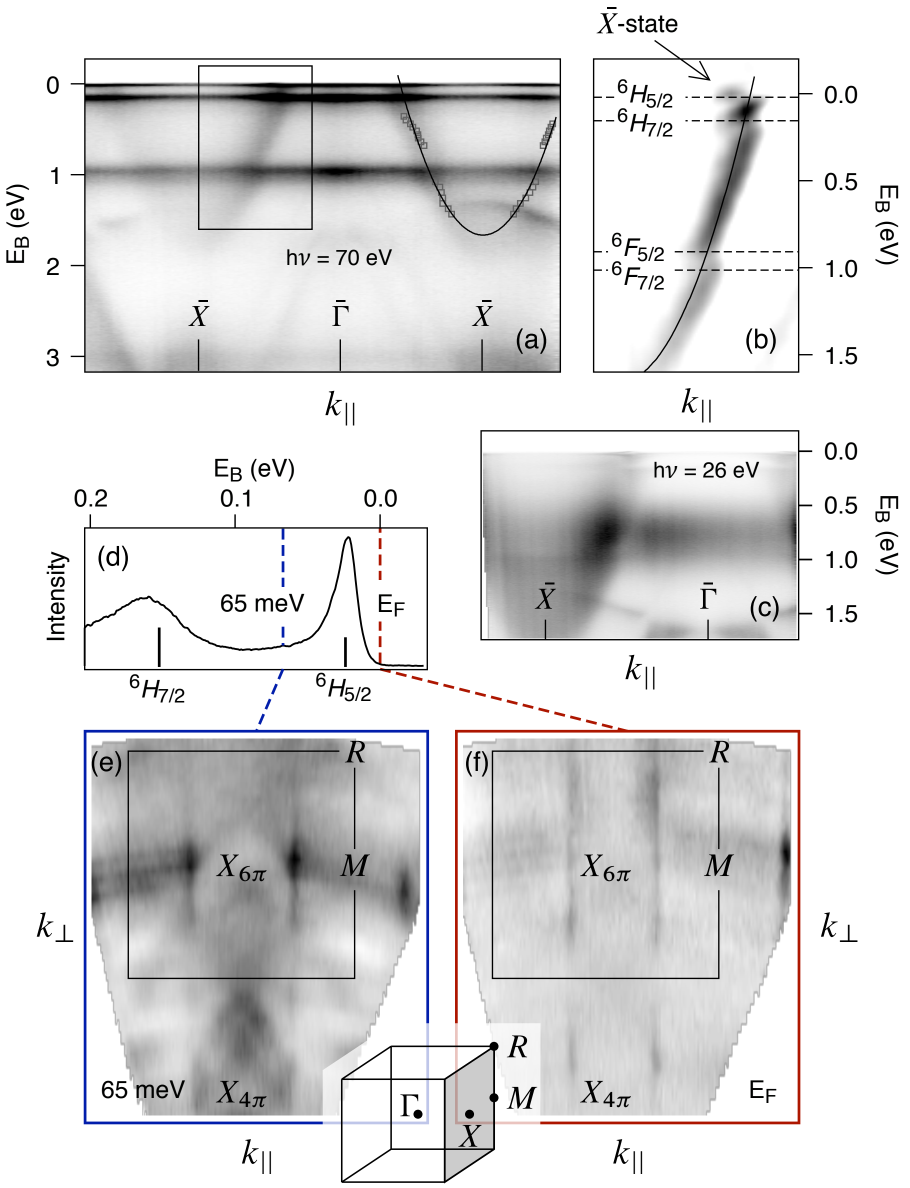

Supplementary Fig. 6. Persistence of two-dimensionality at $\bar{X}$ beyond the bulk hybridization gap. In all previous work [5–11], the d-band at X with $E_{\mathrm{B}} = 1.7$ eV —shown in (a–c) for a B terminated sample along $\bar{\Gamma} - \bar{X}$— has been interpreted as the bulk band that hybridizes with the f-level to yield the bulk band gap. Markers in (a) indicate position of maxima in fits of momentum distribution curves; the solid line is a parabolic fit to these points (markers and solid line horizontally offset by 2π). (b) Second derivative (d^2I/dk^2) of the area marked by the rectangle in (a). Energies of the f-orbital multiplets are indicated by dashed lines. Panel (c) illustrates the $k_\perp$ resolution achieved in photoemission with $h\nu = 26$ eV; $k_\perp \sim 4\pi/a$. The parabola-like dispersion of the $5d$ band around X is filled nearly up to E_{F}. This result shows that three-dimensional features smaller than $\pi/2a$ cannot be resolved under these conditions. Photon energy dependent photoemission results are shown in (e) and (f). In contrast to the studies cited above, we find clear evidence that the Sm $5d$-like feature is not exclusively three-dimensional at binding energies beyond the hybridization gap region: Photon energy dependent measurements unequivocally show the existence of a $k_\perp$-independent state at energies above as well as below the shallowest f-orbital multiplet $(^6H_{5/2})$. The photoemission intensity on the X–M–R plane is shown in (e,f). We have chosen this plane to exclude that a two-dimensional feature appears due to smearing of the intensity from the larger number of X-points on the Γ–X–M plane. While traces of a feature stemming from the three-dimensional d-band around X can be observed below the $^6H_{5/2}$ level in (e), the vertical features in this panel provide clear

evidence of a state that does not depend on the electron wave vector perpendicular to the surface $k_\perp$. Binding energies of the ($k_{||}$, $k_\perp$) maps (e,f) are indicated by dashed lines in (d). Denlinger et al. have also attempted to determine the dimensionality of the electronic structure beyond the f-orbital multiplet [10], but have not found evidence of a two-dimensional state. We attribute this discrepancy to a photoemission matrix element effect, as we find the intensity of the two-dimensional feature to vary strongly with light polarization (it is suppressed with *s*-polarization in the given geometry). Hagiwara et al., on the other hand, have also observed $k_\perp$ independent intensity beyond the hybridization gap in YbB_{12} [12], very similar to our result shown in (e). All results obtained with *p*-polarization.

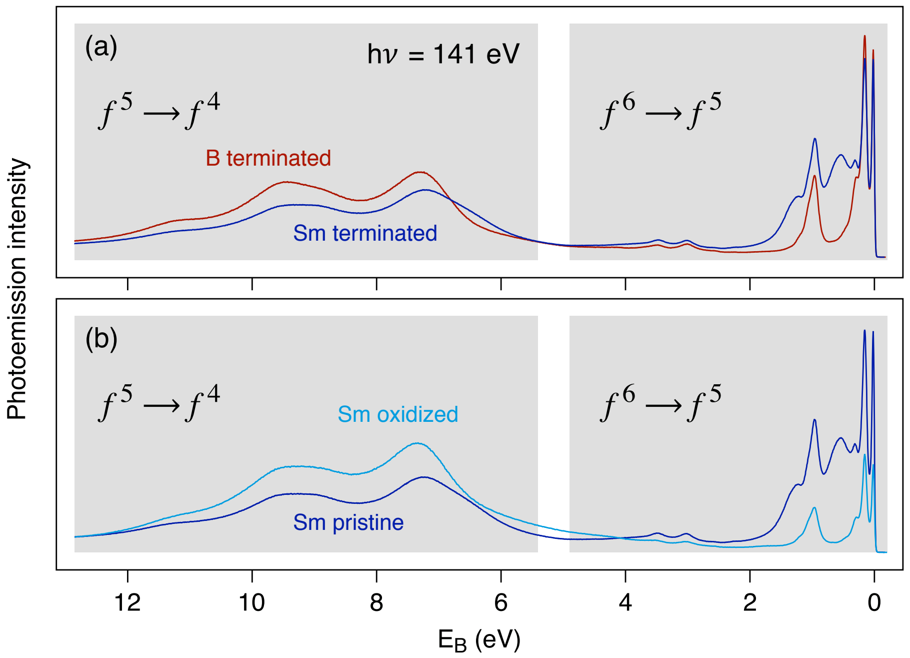

Supplementary Fig. 7. Resonant photoemission from differently terminated surfaces. The photoemission intensity due to Sm in the f^5 initial state configuration is weak compared to the B $2p$ intensity at photon energies below 100 eV. This ratio can be greatly enhanced by using a photon energy near the Sm 4d absorption energy, providing us with a more reliable estimate of the relative weights of f^5 and f^6 configurations. (a) Resonant photoemission ($h\nu$ = 141 eV) spectra of the pristine B (red) and Sm terminated (blue) surfaces. (b) Spectra for the pristine Sm terminated surface (blue) and the Sm_2O_3 covered surface (light blue). Intensity of curves in (a) and (b) is normalized to yield the same integrated intensity; s-polarization. We will interpret the results in terms of the f^5 to f^6 intensity ratios for different terminations: For B termination we propose at least two components with potentially significantly different 4f filling (main text). Sm termination adds a third component in the form of the under-coordinated surface Sm layer. The highest f^6 contribution is found at the Sm terminated sample. We attribute this to the under-coordinated Sm that must be single valent: The intensity of the shallowest f^6 multiplet peak gives rise to the wider feature at a binding energy of approximately 300 meV. In a homogeneous mixed valent system, the f^5 final state would be a component of the ground state and photoemission, as a probe of the energy difference between initial and final states, should yield a maximum at E_F. The other extreme, the spectrum with the highest f^5 initial state share, is observed for the Sm terminated sample oxidized in the ultra-high vacuum residual gas at room temperature for 10 days. In this case we expect the crystal to be capped by a thin Sm_2O_3 (Sm^{3+}) film, corroborated by the strong increase in the intensity of the signal in the f^5 region. The spectrum from the pristine B terminated sample falls between the limiting cases, suggesting the system is mixed valent at both surface and bulk.

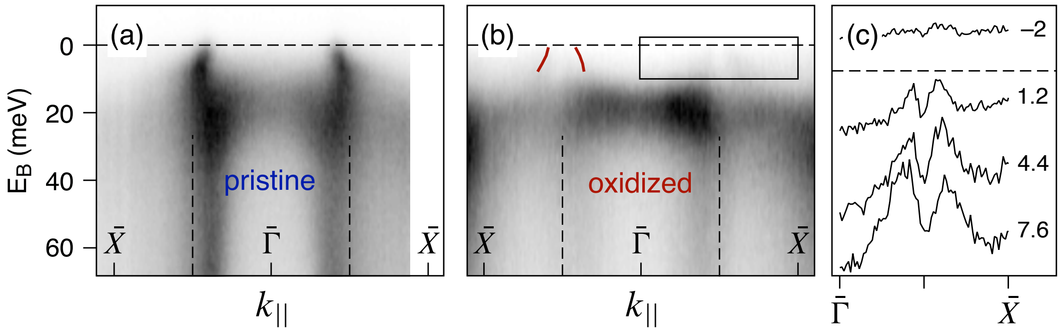

Supplementary Fig. 8. The ultra-thin oxide interface as an approximation of the naturally oxidized sample. We have tried to mimic the termination of the SmB_6 crystal by its natural oxide to link our results to experiments performed on samples exposed to ambient conditions [13, 14]. We have prepared a model of the oxide capped crystal in the following way: It has been reported that the component assigned to under-coordinated Sm at the Sm terminated surface changes readily upon exposure to the residual gas at low temperature [1, 4]. The valency, however, does not change, as the chemical shifts remain in the Sm^{2+} initial state range. Prolonged exposure to the residual gas at room temperature, does change the valency as signaled by an increase in Sm^{3+} intensity at the expense of Sm^{2+}. This effect is illustrated in Supplementary Fig. 7(b). We attribute the increase in the f^5 part of the spectrum that to the Sm sesquioxide, Sm_2O_3. Because the Sm in Sm_2O_3 is entirely trivalent, any f^6 intensity remaining after oxidation must be due to SmB_6 below the ultra-thin oxide layer. We note that the appearance of an originally Sm terminated surface (a) becomes more B termination-like after oxidation (b). The intensity of the $\bar{X}$ state is weakened considerably, but nonetheless sufficient to determine its size. We find that the Fermi wave vector of the $\bar{X}$ state resembles that of the mildly aged B terminated surface [Supplementary Fig. 4(a)]. (a) Photoemission intensity from a pristine Sm terminated $Sm_{0.9}La_{0.1}B_6$ sample. (b) After prolonged (10 d) exposure to the ultra-high vacuum ($\sim 3 \times 10^{-10}$ mbar) residual gas at room temperature. (c) Momentum distribution curves between $\bar{\Gamma}$ and $\bar{X}$ at the indicated binding energies (in meV) [range is also indicated by the dashed rectangle in (b)].

Supplementary Note 1: Umklapp Intensity

We clearly observe the effect of a superstructure with twice the lattice constant on B terminated surfaces [see Figs. 1(a–d) of the main text]. Folded intensity has also been reported by Xu and coworkers [7] and by Jiang, Li and Zhang et al. [6]. The umklapp intensity depends strongly on the photon energy and polarization. This can be seen by comparing Figs. 1(a) and 2(a) from the main text taken with the same photon energy ($h\nu = 31$ eV) but with *s*- and *p*-polarization. In our geometry the umklapp intensity is only observed at photon energies < 40 eV. The observation that the folded intensity can be larger than the original one —as in Fig. 1(a) of the main text— and does not decay rapidly when moving away from the superstructure zone boundary, shows that the superstructure potential does not affect the initial state of the photoemission process. This notion is corroborated by the fact that we do not observe the opening of a gap where the $\bar{X}$ state dispersion meets the folding zone boundary. This can be concluded from Supplementary Fig. 4(b) where we approximate the $\bar{X}$ state on a small energy scale by a linear dispersion. Within this approximation, we would expect the center of the gap to lie within 1 meV from the Fermi level (crossing of the lower pair of lines). No sign of gap opening around this energy can be seen in the photoemission intensity in Fig. 4(a). We therefore suggest that the replicas are due to a superstructure in the covering B layer that affects only the final state in the photoemission process [15]. Paradoxically, the STM studies [2, 16] do not report a superstructure with a twofold periodicity for the areas that provide the best match for B termination on the basis of the ~ 27 meV Sm $4f$ component. We note, however, that the STM measurements have been taken with bias voltages well below the energetic threshold of all B $2p$ state density. The tunneling current therefore consists exclusively of contributions from Sm states. We propose that a twofold structure could appear in appropriately biased (< -1 V) differential conductance images.

The $\bar{X}$ state on the Sm terminated surface displays a less pronounced umklapp feature highlighted in Fig. 1(f) of the main text, possibly caused by the (2×1) structure that both Yee and He et al. [2] and Rößler et al. [16] observe. These areas appear to match Sm termination since they exhibit a shallow differential conductance maximum in good agreement with the ~ 10 meV Sm $4f$ surface component we observe. Both STM groups interpret the (2×1) structure in terms of a missing-row reconstruction. This assignment is not compatible with our results. The chemical sensitivity offered by photoemission spectroscopy suggests that the crystal tends to cleave with chemically pure well-ordered terminations.

Supplementary References

(1) Denlinger, J. D. *et al*. SmB_6 Photoemission: Past and Present. JPS Conf. Proc. 3, 017038 (2014).

(2) Yee, M. M. *et al*. Imaging the Kondo Insulating Gap on SmB_6. Preprint at http://arxiv.org/abs/1308.1085v2 (2013).

(3) Gerken, F. Calculated Photoemission Spectra of the 4*f* States in the Rare-Earth-Metals. J. Phys. F: Met. Phys. 13, 703–713 (1983).

(4) Zhu, Z. H. *et al*. Polarity-Driven Surface Metallicity in SmB_6. Phys. Rev. Lett. 111, 216402 (2013).

(5) Neupane, M. *et al*. Surface electronic structure of the topological Kondo-insulator candidate correlated electron system SmB_6. Nat. Commun. 4, 2991 (2013).

(6) Jiang, J. *et al*. Observation of possible topological in-gap surface states in the Kondo insulator SmB_6. Nat. Commun. 4, 3010 (2013).

(7) Xu, N. *et al*. Surface and bulk electronic structure of the strongly correlated system SmB_6 and implications for a topological Kondo insulator. Phys. Rev. B 88, 121102 (2013).

(8) Xu, N. *et al*. Direct observation of the spin texture in SmB_6 as evidence of the topological Kondo insulator. Nat. Commun. 5, 4566 (2014).

(9) Min, C.-H. *et al*. Importance of Charge Fluctuations for the Topological Phase in SmB_6. Phys. Rev. Lett. 112, 226402 (2014).

(10) Denlinger, J. D. *et al*. Temperature Dependence of Linked Gap and Surface State Evolution in the Mixed Valent Topological Insulator SmB_6. Preprint at http://arxiv.org/abs/1312.6637v2 (2013).

(11) Frantzeskakis, E. *et al*. Kondo Hybridization and the Origin of Metallic States at the (001) Surface of SmB_6. Phys. Rev. X 3, 041024 (2013).

(12) Hagiwara, K. *et al*. Surface Kondo effect and non-trivial metallic state of the Kondo insulator YbB_{12}. Nat. Commun. 7, 1–7 (2016).

(13) Wolgast, S. *et al*. Low-temperature surface conduction in the Kondo insulator SmB_6. Phys. Rev. B 88, 180405 (2013).

(14) Cooley, J. C., Aronson, M. C., Fisk, Z. & Canfield, P. C. SmB_6: Kondo Insulator or Exotic Metal? Phys. Rev. Lett. 74, 1629 (1995). Chen, F. *et al*. Magnetoresistance evidence of a surface state and a field-dependent insulating state in the Kondo insulator SmB_6. Phys. Rev. B 91, 205133–5 (2015).

(15) Anderson, J. & Lapeyre, G. J. Chemisorption-Induced Surface Umklapp Processes in Angle-Resolved Synchrotron Photoemission From W(001). Phys. Rev. Lett. 36, 376–379 (1976).

(16) Rößler, S. *et al*. Hybridization gap and Fano resonance in SmB_6. Proc. Natl. Acad. Sci. 111, 4798–4802 (2014).
