# Supplementary material for: Samarium hexaboride is a trivial surface conductor
Source: Nat Commun. 2018 Feb 6;9:517. doi: 10.1038/s41467-018-02908-7 (PMC5802797; doi:10.1038/s41467-018-02908-7)
Supplement: Supplementary file 2 — Peer Review File [file 41467_2018_2908_MOESM2_ESM.pdf]

Editorial Note: This manuscript has been previously reviewed at another journal that is not operating a transparent peer review scheme. The manuscript was considered suitable for publication without further review at *Nature Communications.*
